# Cell intrinsic role of NF-κB-inducing kinase in regulating T cell-mediated immune and autoimmune responses

**DOI:** 10.1038/srep22115

**Published:** 2016-02-25

**Authors:** Yanchuan Li, Hui Wang, Xiaofei Zhou, Xiaoping Xie, Xiang Chen, Zuliang Jie, Qiang Zou, Hongbo Hu, Lele Zhu, Xuhong Cheng, Hans D Brightbill, Lawren C. Wu, Linfang Wang, Shao-Cong Sun

**Affiliations:** 1 National Laboratory of Medical Molecular Biology, Department of Biochemistry and Molecular Biology, Institute of Basic Medical Sciences, Chinese Academy of Medical Sciences, Peking Union Medical College, Tsinghua University, Beijing, China; 2 Department of Immunology, The University of Texas MD Anderson Cancer Center, 7455 Fannin Street, Box 902, Houston TX 77030, USA; 3 The University of Texas Graduate School of Biomedical Sciences, Houston, TX 77030, USA; 4 State Key Laboratory of Biotherapy, West China Hospital, Si-Chuan University and Collaborative Innovation Center for Biotherapy, Chengdu, 610041, P. R. China; 5 Department of Immunology, Genentech Inc., South San Francisco, CA 94080.

## Abstract

NF-κB inducing kinase (NIK) is a central component of the noncanonical NF-κB signaling pathway. Although NIK has been extensively studied for its function in the regulation of lymphoid organ development and B-cell maturation, the role of NIK in regulating T cell functions remains unclear and controversial. Using T cell-conditional NIK knockout mice, we here demonstrate that although NIK is dispensable for thymocyte development, it has a cell-intrinsic role in regulating the homeostasis and function of peripheral T cells. T cell-specific NIK ablation reduced the frequency of effector/memory-like T cells and impaired T cell responses to bacterial infection. The T cell-conditional NIK knockout mice were also defective in generation of inflammatory T cells and refractory to the induction of a T cell-dependent autoimmune disease, experimental autoimmune encephalomyelitis. Our data suggest a crucial role for NIK in mediating the generation of effector T cells and their recall responses to antigens. Together, these findings establish NIK as a cell-intrinsic mediator of T cell functions in both immune and autoimmune responses.

Transcription factor NF-κB regulates diverse biological processes, including various aspects of immune functions^1^,^2^. NF-κB represents a family of structurally related transcription factors capable of forming homo- and hetero-dimers that bind to the κB enhancer of a large array of target genes. NF-κB activation is mediated by both canonical and noncanonical pathways, which lead to activation of different NF-κB dimers and mediate distinct biological functions^3^,^4^. The noncanonical NF-κB pathway depends on the processing of the NF-κB precursor protein p100 to the mature NF-κB subunit p52. Since p100 also functions as an IκB-like protein, the p100 processing serves to both produce p52 and activate p100-associated NF-κB members^5^.

A central component mediating the activation of noncanonical NF-κB pathway is NF-κB-inducing kinase (NIK), a member of MAP kinase kinase kinase (MAP3K) family 4. NIK gene mutation in both mice and human is associated with severe immune deficiencies^6^. Well-defined functions of NIK and its downstream noncanonical NF-κB pathway include the development of lymphoid organs and maturation of B cells. NIK-deficient mice lack peripheral lymph nodes and have abnormal splenic architecture^6^,^8^. Furthermore, NIK is required for development of thymic epithelial cells, thereby regulating the selection of thymocytes during their development^9^. Thus, some of the abnormal functions of T cells in NIK knockout (KO) mice may be attributed to their impaired selection during development. NIK also regulates the development and maturation of dendritic cells (DCs), suggesting that some of the immune deficiencies associated with NIK deficiency could be due to a defect in antigen presentation.

Given the complexity of NIK function in the development and differentiation of lymphoid organs and immune cells, the study of cell-intrinsic functions of NIK requires NIK conditional KO mice. In particular, the role of NIK in regulating T cell function has been controversial. While some studies suggest a role for NIK in regulating T cell-mediated immunity and autoimmunity, other studies suggest the indirect effect from accessary cells, such as DCs^10^,^11^,^12^. In the current study, we employed conditional KO mice lacking NIK specifically in T cells. We show that NIK has a cell-intrinsic role in regulating the homeostasis and function of T cells. NIK is required for *in vivo* differentiation of inflammatory T cells and the induction of a T cell-dependent autoimmune disease, experimental autoimmune encephalomyelitis (EAE).

## Results

### T cell-specific NIK ablation does not affect thymocyte development

**Figure 1:**
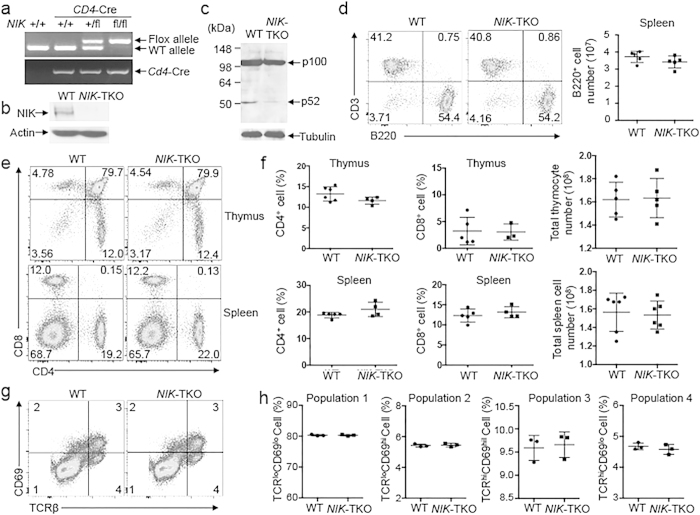
T cell-specific NIK ablation does not affect thymocyte development. (**a**) Genotyping PCR analysis of wildtype (WT) and floxed alleles of *NIK* as well as Cre using tail DNA of the indicated mice. (**b**) Immunoblotting analysis of NIK using whole cell lysates of WT and *NIK*-TKO naïve CD4^+^ T cells treated with 10 μM MG132 for 5 h (to enhance NIK level). (**c**) Immunoblotting analysis of p100 and p52 using whole cell lysates from naïve CD4^+^ T cells stimulated for 48 hours with plate-bound anti-CD3 plus anti-CD28. (**d**) Flow cytometry analyses of B cells (B220^+^CD3^−^) in the spleen of age-matched 6–8 weeks old WT and *NIK*-TKO mice. Data are representative of 3 independent experiments. (**e,f**) Flow cytometry analyses of thymocyte and T cell populations in the thymus and spleen of age-matched (6–8 weeks old) WT and *NIK*-TKO mice based on CD4 and CD8 staining. Data are presented as a representative plot (**e**) and mean ± SD values of multiple mice (**f**, each circle represents a mouse). (**g,h**) Flow cytometry analysis of the surface expression of CD69 and TCRβ on thymocytes from 5 weeks old WT and *NIK*-TKO mice, showing four subpopulations (1, TCR^lo^CD69^lo^; 2, TCR^lo^CD69^hi^; 3, TCR^hi^CD69^hi^; 4, TCR^hi^CD69^lo^) of thymocytes, presented as a representative plot (**g**) and and mean ± SD values of multiple mice (**h**).

Canonical NF-κB plays an important role in regulating development of both conventional T cells and Treg cells^1^. Although global NIK-KO mice have abnormal T-cell selection, it is likely that the impaired development of thymic epithelial cells may contribute to this phenotype. To examine the cell-intrinsic function of NIK in regulating thymocyte development and peripheral T-cell function, we generated NIK T cell-conditional KO (*NIK*-TKO) mice by crossing NIK-flox mice with *CD4*-Cre mice (Fig. 1a). Since NIK level is extremely low due to its continuous degradation^13^, we treated the cells with a proteasome inhibitor, MG132, for NIK detection by immunoblot. NIK was detected in wildtype T cells, but not in *NIK*-TKO T cells, suggesting efficient ablation of NIK by the CD4-Cre (Fig. 1b). Consistently, the *NIK*-TKO T cells had a defect in anti-CD3/anti-CD28-stimulated p100 processing (generation of p52) (Fig. 1c). In contrast to the severe reduction of B cell numbers in the global *NIK*-KO mice, the *NIK*-TKO mice had normal frequency of B cells in the spleen (Fig. 1d). The T cell-specific NIK deficiency also did not have an obvious effect on thymocyte development, as evidenced by the comparable frequency of different subpopulations of thymocytes, including CD4^−^CD8^−^ double-negative (DN), CD4^+^CD8^+^ double-positive (DP), CD4^+^CD8^−^ and CD8^+^CD4^−^ single-positive (SP) cells (Fig. 1e). Consistently, the *NIK*-TKO and wildtype control mice had comparable frequency of peripheral CD4^+^ and CD8^+^ T cells in the spleen (Fig. 1e,f). Thymocyte development from DP to SP stages involves positive selection, a process that is associated with upregulation of surface TCRβ and CD69^14^,^15^. Flow cytometry analysis revealed that the wildtype and NIK-deficient thymocytes contained similar percentages of TCR^lo^CD69^lo^ (preselection), TCR^lo^CD69^hi^ (transitional), TCR^hi^CD69^hi^ (postselection), and TCR^hi^CD69^lo^ (mature) populations (Fig. 1g,h). Thus, NIK expression in T-cell compartment is dispensable for thymocyte development.

### NIK has a cell-intrinsic role in regulating the homeostasis of peripheral T cells and Treg cells

**Figure 2:**
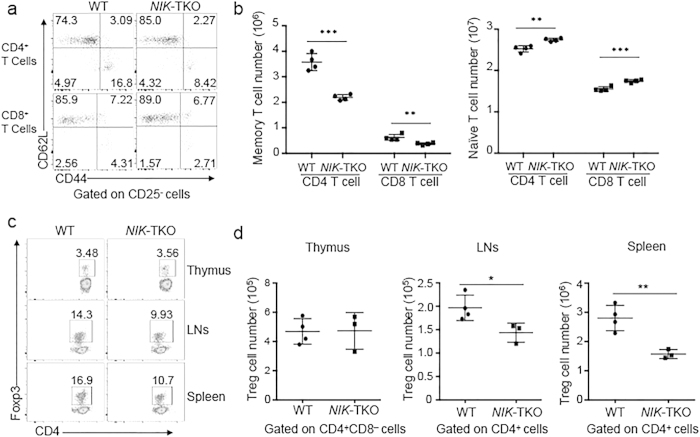
A cell-autologous role for NIK in regulating T cell homeostasis *in vivo*. (**a,b**). Frequency (**a**) and absolute number (**b**) of naïve (CD44^lo^CD62L^hi^) and memory (CD44^hi^CD62L^lo^) CD4^+^ or CD8^+^ T cells among total splenocytes from age-matched 6–8 weeks old WT and *NIK*-TKO. The CD25^+^ cell population was excluded to avoid influence from Treg cells. Numbers in quadrants indicate percentage of the cell populations. Data are presented as a representative plot (**a**) and mean ± SD values of multiple mice (**b**, each circle represents a mouse). (**c,d**) Flow cytometry analyses of Treg cell frequency (**c**) and absolute numbers (**d**) in CD4^+^ mature thymus (CD4^+^CD8^−^) and peripheral CD4^+^ T cells in the lymph nodes (LNs) and spleen of age-matched (6–8 weeks old) WT and *NIK*-TKO mice. Data are presented as a representative plot (**c**) mean ± SD values of multiple mice (**d**, each circle represents a mouse).

The maintenance of naïve and memory T cells in the peripheral lymphoid organs involves homeostatic signaling pathways stimulated by both cytokines and self-peptide-MHC ligands for TCR^16^. To assess the cell-intrinsic function of NIK in regulating T-cell homeostasis, we analyzed the frequency of naïve and memory T cells in the peripheral lymphoid organs. As expected, the splenocytes of wildtype mice contain both naïve and memory T cells, characterized by the CD44^lo^CD62L^hi^ and CD44^hi^CD62L^lo^ surface markers, respectively. Interestingly, although *NIK*-TKO and wildtype mice had similar frequency of total CD4^+^ and CD8^+^ T cells in the spleen (Fig. 1e,f), the *NIK*-TKO mice had significantly reduced frequency and number of the memory population and concomitantly increased naïve population of T cells in the spleen (Fig. 2a,b). These results suggest a role for NIK in regulating the homeostasis of memory like T cells.

We next examined whether the NIK deficiency altered the generation or maintenance of regulatory T (Treg) cells. In the thymus, the *NIK*-TKO mice and wildtype control mice had a similar frequency of the Foxp3^+^ Treg cells, suggesting a dispensable role for NIK in regulating Treg production (Fig. 2c,d). However, the *NIK*-TKO mice had a significantly lower frequency of Treg cells in the peripheral lymphoid organs, lymph nodes and spleen (Fig. 2c,d). Thus, NIK expression in T-cell compartment is important for the maintenance of both memory-like T cells and Treg cells in the periphery.

### NIK is dispensable for naïve T-cell activation *in vitro*

**Figure 3:**
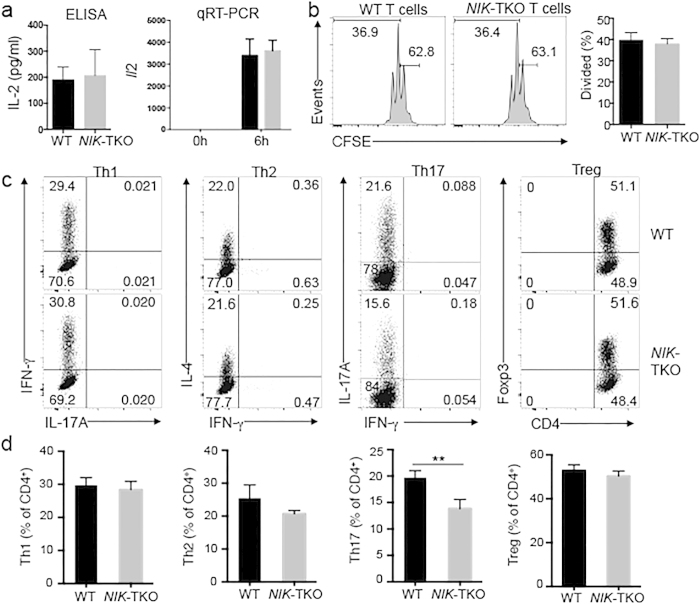
NIK regulates Th17 differentiation but is dispensable for naïve CD4 T-cell activation *in vitro*. (**a**) ELISA and qRT-PCR analyses of IL-2 expression in naïve CD4^+^ T cells (CD44^lo^CD62L^hi^) purified from splenocytes of age-matched (6–8 weeks old) WT and *NIK*-TKO mice, stimulated for 6 h (for qRT-PCR) or 48 h (for ELISA) with plate-bound anti-CD3 plus anti-CD28 antibodies. Data are representative of 3 independent experiments (mean ± SD). (**b**) Naïve CD4^+^ T cells from WT and *NIK*-TKO mice were labeled with CFSE and stimulated for 72 hours with plate-bound anti-CD3 plus anti-CD28 antibodies. Cell proliferation was measured by flow cytometry and determined based on CFSE dilution during cell division. Data are representative of 3 independent experiments. (**c,d**) Naïve splenic CD4^+^ T cells of WT and *NIK*-TKO mice were stimulated for 3 d with plate-bound anti-CD3 plus anti-CD28 under Th1, Th17, or Treg conditions or 5d under Th2 conditions as described in Materials and Methods. Flow cytometry was performed to measure the frequency of the indicated subsets of effector T cells based on intracellular cytokine staining or the Foxp3^+^ Treg cells. Data are representative (**d**) and mean ± SD values of 3 independent experiments.

The important role of NIK in regulating T-cell homeostasis *in vivo* prompted us to examine whether NIK is required for T-cell activation. We purified naïve CD4^+^ T cells from young adult mice and stimulated them *in vitro* using monoclonal antibodies for TCR (anti-CD3) and CD28 (anti-CD28). As expected, wildtype T cells produced the T cell growth factor IL-2 in response to *in vitro* stimulation (Fig. 3a). NIK ablation in T cells did not appreciably affect this important molecular event of T cell activation (Fig. 3a). The NIK deficiency also did not influence the induction of *Il2* mRNA, as revealed by a real-time quantitative RT-PCR (qRT-PCR) assay (Fig. 3a). Furthermore, the NIK-deficient and wildtype naïve T cells displayed a similar level of proliferative ability when stimulated *in vitro* with anti-CD3 and anti-CD28 antibodies (Fig. 3b).

Following activation, naïve CD4^+^ T cells differentiate into various subsets of effector T cells, including the interferon-gamma (IFN-γ)-producing T helper (Th)1 cells, IL-4-producing Th2 cells, IL-17A-producing Th17 cells, as well as the Foxp3^+^ Treg cells^17^. The CD4^+^ T cell differentiation is controlled by both the TCR signal and signals stimulated by various cytokines. We examined the role of NIK in regulating T cell differentiation using an *in vitro* model system. The T cell-specific ablation of NIK had little or no effect on the induction of Th1, Th2, or Treg cells (Fig. 3c). However, the induction of Th17 cells was partially inhibited in the NIK-TKO T cells (Fig. 3c,d). These results suggest that NIK is dispensable for *in vitro* activation of T cells but plays a role in regulating Th17 cell differentiation.

### NIK regulates antigen-stimulated *in vivo* T cell differentiation and recall responses

**Figure 4:**
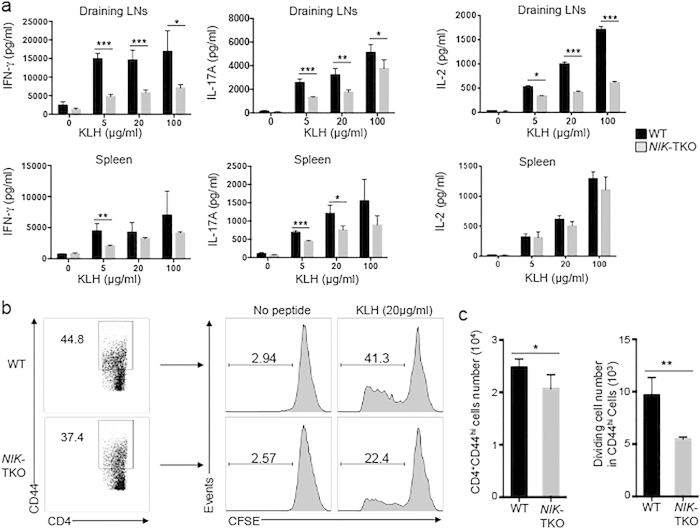
NIK regulates generation of antigen-specific effector T cells *in vivo*. (**a**) Age-matched (6–8 weeks old) WT and *NIK*-TKO mice were immunized with KLH in CFA. A week later, draining lymph node cells and splenocytes were harvested and restimulated with KLH for 3 days followed by detecting IFN-γ and IL-17 A in the supernatant by ELISA. (**b,c**) KLH-stimulated draining lymph cells from **a** were labeled with CFSE to assess KLH-stimulated proliferation of CD4^+^CD44^+^ T cells. After 4 days of stimulation with KLH (20 μg/ml), the percentage (**b**) and absolute numbers (**c**) of dividing cells (CFSE^low^) were quantified by flow cytometry and presented as a representative plot (**b**) and means ± SD values of multiple mice (**c**, 4 mice/group).

To examine the role of NIK in regulating antigen-stimulated T-cell responses *in vivo*, we immunized wildtype and NIK-TKO mice with a protein antigen, Keyhole limpet hemocyanin (KLH). After a week of immunization, we isolated draining lymph node cells and splenocytes for *in vitro* restimulation of the antigen-specific effector T cells with KLH. Both the draining lymph node and the spleen of immunized mice contained antigen-specific Th1 and Th17 effector T cells, which produced IFN-γ and IL-17A upon *in vitro* restimulation with KLH (Fig. 4a). Importantly, compared to the wildtype mice, the *NIK*-TKO mice had a significantly lower level of Th1 and Th17 responses, particularly in the draining lymph nodes where the majority of effector T cells reside (Fig. 4a). It appeared that NIK was crucial for antigen-stimulated recall responses of the effector T cells, since the NIK-deficient T cells from the draining lymph nodes, but not the spleen, were deficient in IL-2 production in response to KLH stimulation (Fig. 4a). In further support of this possibility, the NIK-deficient CD44^hi^ effector T cells derived from the draining lymph nodes of KLH-immunized mice had attenuated proliferation when restimulated *in vitro* with KLH (Fig. 4b,c). Thus, NIK appears to regulate the *in vivo* differentiation of Th1 and Th17 cells and the recall responses of the effector T cells.

### NIK has a T cell-intrinsic function in regulating EAE pathogenesis

**Figure 5:**
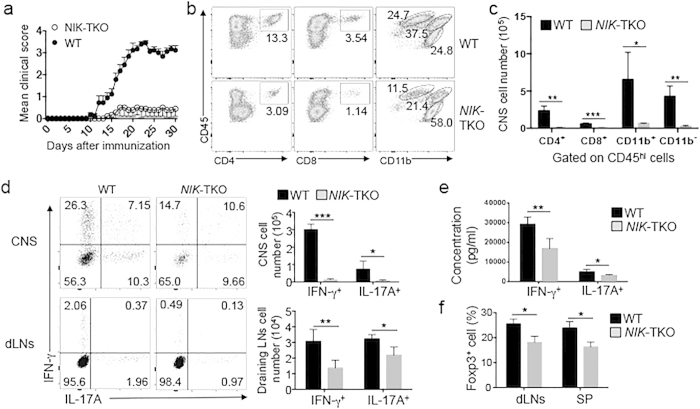
NIK has a T cell-intrinsic role in regulating EAE. (**a**) Mean EAE clinical scores of age- and sex-matched WT and *NIK*-TKO mice (n = 9 mice per group) immunized with MOG_35–55_ for EAE induction. (**b, c**) Flow cytometry analysis of CNS-infiltrating immune cells (CD45^hi^) in day 16 MOG_35–55_-immunized EAE mice (n = 3 mice per group). Data are presented as a representative plot showing the percentage of gated cell populations (**b**) and summary graph of absolute cell numbers (**c**). (**d**) Flow cytometry analysis of percentage (left) and absolute number (right) of CD4^+^IFN-γ^+^ Th1 and CD4^+^IL-17 A^+^ Th17 cells in the CNS (brain and spinal cord) and draining lymph nodes (dLNs) of MOG_35–55_-immunized mice (n = 3 mice per group) on day 16 after immunization. (**e**) ELISA analyses of IFN-γ and IL-17 A in supernatants of draining lymph node cells isolated from day 16 MOG_35–55_-immunized mice and stimulated *in vitro* for 72 h with MOG_35–55_ (20 μg/ml). **(f)** Flow cytometry analyses of the frequency of the Treg cells (CD4^+^Foxp3^+^) in MOG_35–55_-immunized EAE mice on day 32 after immunization. Data are presented as mean ± SD values of multiple mice (each circle represents a mouse). Data are representative of 3 independent experiments.

To further examine the T cell-intrinsic role of NIK in regulating T cell differentiation and effector function *in vivo*, we employed a T cell-dependent autoimmunity model, experimental autoimmune encephalomyelitis (EAE)^18^. EAE is an animal model of the neutroinflammatory disease multiple sclerosis, and the development of EAE involves peripheral priming of T cells by an central nervous system (CNS)-specific autoantigen and the subsequent migration of autoimmune effector T cells to the CNS^18^,^19^,^20^. In particular, both Th1 and Th17 subsets of CD4^+^ effector T cells play an important role for the pathogenesis of EAE^20^. We induced EAE by immunizing mice with a myelin-specific autoantigen, myelin oligodendrocyte glycoprotein (MOG) peptide MOG_35–55_. As expected, immunization of wildtype mice led to the development of severe EAE clinical scores (Fig. 5a). In sharp contrast, the NIK-TKO mice were largely refractory to the induction of EAE (Fig. 5a). Flow cytometric analysis revealed that the NIK-TKO mice had substantially reduced frequencies of CNS-infiltrating CD4^+^ and CD8^+^ T cells as well as CD11b^+^CD45^hi^ cells, known to contain infiltrating monocytes and activated microglia (Fig. 5b). Concomitantly, the frequency of the resting CNS-resident microglial cells, characterized by CD11b^+^CD45^lo^ surface markers, was greatly increased in the *NIK*-TKO mice (Fig. 5b). Since the *NIK*-TKO mice had drastically reduced total CD45^hi^ infiltrating immune cells, the absolute number of CNS-infiltrating T cells and monocytes in the EAE-induced NIK-TKO mice was strikingly lower than that in the EAE-induced wildtype mice (Fig. 5c).

To examine the role of NIK in regulating the production of inflammatory effector T cells, we analyzed the number of IFN-γ-producing Th1 and IL-17A-producing Th17 cells in the CNS and draining lymph nodes of the MOG-immunized mice. Compared with the wildtype mice, the NIK-TKO mice had a much smaller number of Th1 and Th17 cells in the CNS (Fig. 5d). While this result was obviously due to the reduction in the number of total CD4^+^ T cells in the *NIK*-TKO CNS (Fig. 5c), the NIK-TKO mice also had a reduced number of Th1 and Th17 cells in the draining lymph node (Fig. 5d). The lymph node T cells isolated from MOG_35–55_-immunized *NIK*-TKO mice also produced lower amounts of secreted IFN-γ and IL-17A relative to lymph node T cells derived from MOG_35–55_-immunized wildtype mice, following *in vitro* restimulation with MOG_35–55_ peptide (Fig. 5e). These results, along with the result shown in Fig. 4a, suggest that NIK plays a role in mediating the production of Th1 and Th17 subsets of CD4^+^ effector cells *in vivo*.

Since Treg cells are involved in the regulation of EAE pathogenesis^21^, we examined the effect of NIK deficiency in T cells on Treg induction during EAE. Compared to the wildtype mice, the *NIK*-TKO mice had reduced frequency of Treg cells in both the draining lymph node and the spleen (Fig. 5f). This result, which was in line with the Treg cell reduction under homeostatic conditions (Fig. 2c,d), suggests that the resistance of *NIK*-TKO mice to EAE induction is not due to overproduction of Treg cells but rather results from a defect in effector T cell generation.

### NIK ablation in T cells attenuates T cell responses to bacterial infections

**Figure 6:**
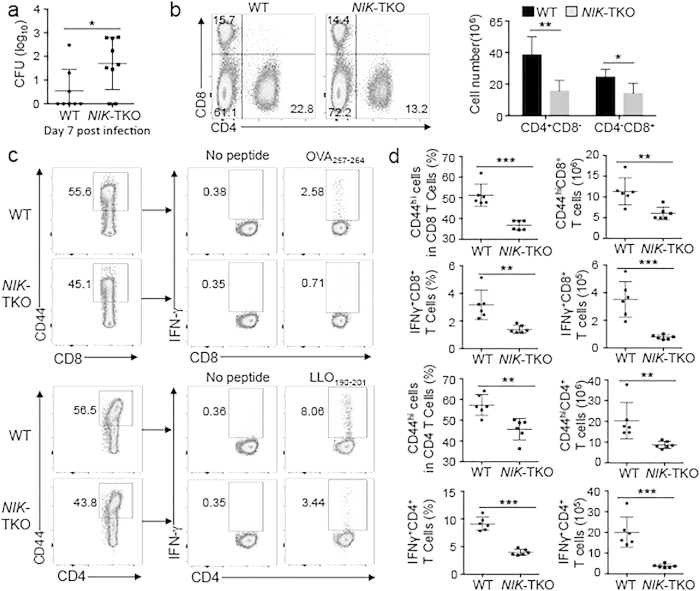
NIK is required for T-cell responses to bacterial infection. (**a**) L. monocytogenes titer in the liver of age-matched wildtype (WT) and NIK-TKO mice infected with LM-OVA (day 7 of infection). (**b**) Flow cytometry analyses of the percentage (left) and absolute number (right) of CD4^+^ and CD8^+^ T cells in the spleen of WT and *NIK*-TKO mice infected with LM-OVA for 7 days. Data are presented as a representative graph (left) and summary graph (right). (**c,d**) ICS and flow cytometry analyses of IFN-γ-producing CD4^+^ and CD8^+^ T cells in the spleen of wildtype (WT) and *NIK*-TKO mice infected with LM-OVA for 7 days. Splencotyes were restimualted for 5 h with LLO_190–201_ or OVA_257–264_ followed by treatment with monesin for 1 hour prior to the analyses, and data are presented as a representative plot (**c**) and a summary graph (**d**).

T cells, particularly IFN-γ-producing Th1 and CD8^+^ T cells, play an important role in host defense against infections by the intracellular bacterial pathogen Listeria monocytogenesis (L. monocytogenesis)^22^,^23^. We examined the T cell-intrinsic role of NIK in mediating immune responses against L. monocytogenesis by infecting wildtype and NIK-TKO mice with a modified *L. monocytogenes* strain encoding chicken ovalbumin (LM-OVA)^24^. Compared to the wildtype mice, the NIK-TKO mice had a significantly higher L. monocytogenes load in the liver, indicating compromised immune response (Fig. 6a). Consistently, the spleens of the infected NIK-TKO mice had a reduction in the frequency and absolute number of CD4^+^ and CD8^+^ T cells (Fig. 6b). The frequency and absolute number of CD44^hi^ CD4^+^ and CD8^+^ T cells, representing effector T cells, were also substantially lower in the spleen of NIK-TKO mice (Fig. 6c,d). Since IFN-γ-producing CD4^+^ and CD8^+^ T cells play a crucial role in host defense against L. monocytogenes infection, we examined the frequency of antigen-specific effector T cells in the infected mice based on *in vitro* restimulation by antigens. As expected, a proportion of IFN-γ-producing CD8^+^ and CD4^+^ effector T cells was detected in the splenocytes of wildtype mice upon *in vitro* restimulation with an MHC class I-restricted OVA peptide (OVA_257–264_) and an MHC class II-restricted listeriolysin O peptide (LLO_190–201_), respectively (Fig. 6c). Importantly, the frequency and absolute number of the IFN-γ-producing antigen-specific CD8^+^ and CD4^+^ effector T cells were profoundly reduced in the *NIK*-TKO mice (Fig. 6c,d). These results suggest a T cell-intrinsic role for NIK in regulating *in vivo* T cell responses to bacterial infections.

## Discussion

The role of NIK in regulating immune responses has been complicated by the essential role of NIK in regulating the development of lymphoid organs, which are required for T cell selection in the thymus and peripheral lymphocyte activation. In the present study, we studied the T cell-intrinsic functions of NIK in the regulation of immune and autoimmune responses. Since NIK functions in stromal cells to regulate lymphoid organ development, the *NIK*-TKO mice did not display any obvious abnormalities in the development of lymphoid organs. The T cell-specific ablation of NIK also did not affect the development of conventional T cells or Treg cells in the thymus. This result suggests that NIK expression in T cells is not required for thymocyte development or thymic Treg production, which is consistent with the previous finding that NIK functions in thymic epithelial cells to regulate thymocyte selection and Treg development^9^,^25^.

Our present work revealed an important T cell-intrinsic role for NIK in regulating homeostasis of T cells, particularly the memory like T cell and Treg cell populations, in the peripheral lymphoid organs. T cell homeostasis is a mechanism that maintains the peripheral pools of mature T cells. This mechanism relies on weak TCR signals, triggered by self-peptide/MHC ligands, and the common γ-chain cytokine IL-7^26^. In addition, the homeostasis of memory-like T cells also requires costimulatory signals from TNF receptor (TNFR) superfamily members, such as OX40 and CD30^27^,^28^. We have previously shown that NIK is required for the induction of noncanonical NF-κB activation in T cells by the TCR and CD28 signals^29^. NIK also mediates T cell costimulation by OX40 and likely additional members of the TNFR family of costimulatory molecules cell subsets^11^,^30^. Thus, NIK may regulate T cell homeostasis via signaling pathways from both the TCR and TNFRs.

Our data suggest that NIK is dispensable for the initial activation of naïve T cells. This finding is consistent with the slow kinetics of NIK induction and noncanonical NF-κB activation along with T cell activation^29^. It is likely that canonical NF-κB plays a major role in mediating T cell activation. Activated T cells are induced to express various TNFR family members that mediate induction of NIK and noncanonical NF-κB signaling, which in turn may contribute to the generation and subsequent function of effector T cells. We found that NIK-TKO mice had a severe defect in producing antigen-specific effector T cells in response to challenge by a protein antigen, KLH, and the CNS-specific self-antigen MOG as well as by the bacterium *L. monocytogenes*. NIK deficiency only moderately inhibited the induction of Th17 cells *in vitro* in a system that involves T cell activation by anti-CD3 and anti-CD28 antibodies. One of the differences between the *in vitro* and *in vivo* systems is that the *in vivo* system more critically involves the contribution of TNFR members, whose ligands are abundantly expressed on antigen-presenting cells. Our *in vivo* model of studies clearly demonstrated a role for NIK in regulating the generation of different subsets of effector T cells, particularly the Th1 and Th17 subsets of inflammatory T cells. In addition, NIK appeared to play a role in the recall responses of antigen-specific effector T cells.

Using conventional NIK-KO mice, we and others have previously shown that NIK regulates EAE induction^10^,^12^. However, how NIK regulates EAE pathogenesis has been in debate, because NIK functions in different cell types including DCs and B cells, which are also involved in the pathogenesis of EAE^10^,^12^. Although bone marrow and T cell transfer studies have been performed, these studies were not conclusive. Using T cell-conditional NIK KO mice, we have now provided definitive evidence for a T cell-intrinsic function of NIK in regulating the effector functions of Th1 and Th17 cells and induction of EAE. A previous study proposes that NIK functions in DCs, but not in T cells, in the regulation of EAE induction, since transgenic expression of NIK in DCs rescues the defect of the NIK-deficient mice in EAE induction^12^. We believe that the discrepancy between our present work and the previous study is due to the use of different mouse models. In particular, the expression of NIK in NIK transgenic mice is extremely high, as opposed to the low physiological level of NIK expression. Furthermore, although NIK transgene is mainly expressed in DCs, the transgenic mice also have a low level of NIK expression in other cells including T cells, thus complicating the phenotype^12^. Nevertheless, the previous studies, together with our present work, suggest that NIK may function in both T cells and DCs to regulate inflammatory T cell responses. Future studies will confirm the DC-specific function of NIK using DC-conditional NIK KO mice.

## Methods

### Mice

The *NIK*-flox mice (on C57BL/6 background), provided by Genentech, were generated using LoxP system targeting exon 2 of the *NIK* gene^31^. To create T cell-conditional NIK KO (*NIK*-TKO) mice, the *NIK*-flox mice were crossed with *Cd4*-Cre transgenic mice (B6 genetic background, Jackson Laboratories). Age-matched NIK-TKO (*NIK*^flox/flox^*Cd4*-Cre) and NIK WT control (*NIK*^+/+^*Cd4*-Cre) mice were used for experiments. Mice were genotyped by PCR analysis of DNA obtained from tail tissue. The genotyping primers were as follows: NIK Forward, 5′-ATCAAGCTGGCCCTTAACCT-3′; and reverse, 5′-CAAGGAGTTCTTGTTTCCCAG-3′. CD4-Cre Forward, 5′-CCCAACCAACAAGAGCTC-3′; and reverse, 5′-CCCAGAAATGCCAGATTACG-3′.

The mice were maintained in a specific pathogen-free facility, and the animal experiments were performed in accordance with protocols approved by the Institutional Animal Care and Use Committee of the University of Texas MD Anderson Cancer Center.

### Antibodies and reagents

Functional-grade antibodies for CD3 (145-2C11), CD28 (37.51) and IFN-γ (XMG1.2) were from eBioscience, and the anti-IL-4 antibody (11B11) was from National Cancer Institute Preclinical Repository. Fluorescence-labeled antibodies for CD3 (17A2), CD4 (RM4-5), CD8 (53-6.7), CD11b (M1/70), CD44 (IM7), CD45 (30-F11), B220 (RA3-6B2), CD62L (MEL-14), CD69 (H1.2F3), TCRβ (H57-597), Foxp3 (FJK-16s), IL-17A (eBio17B7), and IFN-γ (XMG1.2) were purchased from eBioscience. Antibodies for mouse NIK (H248, 1:1,000) and Tubulin (TU-02, 1:2000) were from Santa Cruz Biotechnology. Anti-Actin (C-4, 1:10,000) was from Sigma, and anti-p100/p52 (TB4, 1:8,000) was from National Cancer Institute Preclinical Repository. CellTrace™ CFSE Cell Proliferation Kit was from Thermo Fisher Scientific. The recombinant mouse cytokines IL-2, IL-4, IL-6, IL-12, and TGF-β were from R&D systems. MG132 was from Cayman Chemical.

### Immunoblot (IB) Analysis

Naïve CD4^+^ T cells were stimulated for 48 h with plate-bound anti-CD3 and anti-CD28 antibodies and lysed in radioimmunoprecipitation assay (RIPA) buffer for IB analysis of p100 processing to p52. For NIK IB analysis, the T cells were activated as indicated above and then further expanded in medium supplemented with IL-2 (10 units/mL) for 72 h. Since NIK is constantly degraded^32^, a proteasome inhibitor, MG132 (10 μM), was added to the cell culture during the last 5 h to further enhance the level of NIK. Whole-cell lysates were subjected to immunoprecipitation (IP) using anti-NIK antibody followed by detecting the precipitated NIK by IB (using anti-NIK) as described^32^.

### Flow cytometry, cell sorting and intracellular cytokine staining (ICS)

Flow cytometric analyses and cell sorting were performed^33^ using a LSRII FACSFortessa (BD) and FACSAria (BD), respectively. For ICS, cells were stimulated with 50 ng/ml PMA plus 750 ng/ml ionomycin for 6 h in the presence of 10 μg/ml protein transport inhibitor monensin, and the fixed cells were incubated with the indicated antibodies and subjected to flow cytometry.

### CD4 T-cell isolation and differentiation assays

Total CD4^+^ T cells were isolated from the spleen and lymph nodes using a total CD4^+^ T cell isolation kit (Miltenyi Biotec). Enriched CD4^+^ T cells were separated into naïve CD4^+^ and naïve CD8^+^ T cells by flow cytometric cell sorting based on CD4^+^CD44^lo^CD62L^hi^ and CD8^+^CD44^lo^CD62L^hi^ surface markers, respectively. Purified naïve CD4 T cells were stimulated with plate-bound anti-CD3 (1 μg/ml) and anti-CD28 (1 μg/ml) antibodies under Th1 (10 ng/mL IFN-γ, 10 ng/mL IL-12, 10 μg/mL anti–IL-4), Th2 (10 ng/mL IL-4, 10 μg/mL anti–IFN-γ), Th17 (20 ng/mL IL-6, 5 ng/mL TGF-β, 10 μg/mL anti–IL-4, 10 μg/mL anti–IFN-γ), or Treg (10 units/mL IL-2, 5 ng/mL TGF-β) conditions. After 3 days (Th1, Th17, Treg) or 5 days (Th2) of differentiation, the differentiated T cells were re-stimulated for 6 h with PMA and ionomycin in the presence of the monensin, followed by intracellular staining of IFN-γ, IL-17, IL-4, and Foxp3 to quantify the frequency of Th1, Th17, Th2, and Treg cells.

### CFSE Staining

Cells were stained with CFSE to a final 5 μM concentration with PBS, and immediately incubated for 5 minutes. Staining was stopped by washing the cells with ice cold RPMI-1640 with 10% FCS and incubated in ice for 5 minutes. Cells then were washed twice in culture media. After that, cells were cultured in indicated condition.

### Immunization and T cell recall response assays

Age-matched 6–8 weeks old WT and *NIK*-TKO mice were immunized s.c. (at the base of tail) with Keyhole Limpet Hemocyanin (KLH, 0.5 mg/ml) emulsified in CFA (0.5 mg/ml) at a dose of 100 μl per mouse. Seven days after immunization, these mice were sacrificed and analyzed individually. To analyze the role of NIK in regulation of T cell responses *in vivo*, splenocytes and draining lymph node cells from KLH-immunized mice were cultured in 96-well plates with or without the antigen KLH. After 3 days, ELISA was performed using cell supernatant to measure cytokines.

### Induction and evaluation of EAE

EAE was induced by injecting mice s.c. (in the back region) with MOG_35–55_ peptide (200 μg/mouse) in CFA containing 5 mg/ml heat-killed Mycobacterium tuberculosis (H37Ra strain; BD Diagnostics, Franklin Lakes, NJ). On the day of immunization and 48 h later, the mice were also injected i.v. with pertussis toxin (200 ng/mouse; List Biological Laboratories, Campbell, CA) in PBS. 7 days after the first immunization, mice were immunized with a second dose of MOG_35–55_ peptide, and then examined daily for EAE disease symptoms, which were scored using a standard method^34^: 0, no disease symptoms; 1, limp tail or hind limb weakness (not both); 2, with both a limp tail and hind limb weakness; 3, partial paralysis of hind limbs; 4, complete hind limb paralysis; and 5, moribund (death caused by EAE). 0.5 gradations are utilized for scores falling between two of the above criteria.

### Isolation of CNS mononuclear cells

Single cell suspensions were prepared by smashing brain and spinal cord tissues from EAE mice with a syringe and nylon mesh. The cells were collected by centrifugation and resuspended in 10 ml of 37% percoll (made in sterile PBS). After spinning 20 minutes at 4 °C (2000 × g), upper phase was removed and the mononuclear cells, contained in the pellet in the bottom of the tube, were collected. Cells were washed and treated with red blood cell lysing buffer (Sigma) to lyse erythrocytes and then resuspended in complete media.

### L. monocytogenes infection

Age-matched wildtype and *NIK*-TKO mice were infected (i.v.) with a recombinant *L. monocytogenes* expressing a truncated OVA protein (LM-OVA; provided by Dr. Hao Shen via DMX Inc) (1 × 10^5^ CFU per mouse). After 7 days, the infected mice were sacrificed for analyzing bacterial load in the liver and T cells in the spleen. For detecting antigen-specific CD8^+^ effector T cells, the splenocytes were restimulated with the MHC class I-restricted OVA_257–264_ peptide SIINFEKL (15 μg/mL) for 5 h followed by treatment with monesin for 1 hour and analysis of IFN-γ production by ICS and flow cytometry. CD4^+^ effector T cells were analyzed similarly except for the stimulation of splenocytes with the MHC class II-restricted LLO_190–201_ peptide NEKYAQAYPNVS (15 μg/mL).

### Enzyme-linked immunosorbent assay (ELISA)

After the indicated stimulation, cell culture supernatants were collected for ELISA assays according to the manufacturer’s instructions (eBioscience).

### Real-time quantitative RT-PCR

Total RNA was isolated from T cells using TRI reagent (Invitrogen) and subjected to cDNA synthesis using MMLV reverse transcriptase (Invitrogen) and oligo (dT) primers. Real-time quantitative PCR (qRT-PCR) was performed using iCycler Sequence Detection System (Bio-Rad, Hercules, CA) and iQ SYBR Green Supermix (Bio-Rad). The expression of individual genes was calculated by a standard curve method and was normalized to the expression of *β-Actin* (Actb). The primers used in qRT-PRC assays are shown below. *β-Actin* forward, 5′-CGTGAAAAGATGACCCAGATCA-3′; and reverse, 5′-CACAGCCTGGATGGCTACGT-3′. *mIL-2* forward, 5′-CCTGAGCAGGATGGAGAATTACA-3′; and reverse, 5′-TCCAGAACATGCCGCAGAG-3′.

### Statistical analysis

Two-tailed unpaired t test statistical analysis was performed using Prism software. The p values < 0.05 were considered significant, and the level of significance was indicated as *p < 0.05, **p < 0.01, and ***p < 0.001.

## Additional Information

**How to cite this article**: Li, Y. *et al*. Cell intrinsic role of NF-κB-inducing kinase in regulating T cell-mediated immune and autoimmune responses. *Sci. Rep.* **6**, 22115; doi: 10.1038/srep22115 (2016).
